# Co-occurring mental disorders in treatment admissions for substance use disorders in Maryland

**DOI:** 10.1186/1940-0640-10-S1-A42

**Published:** 2015-02-20

**Authors:** Patience Moyo, Ting-Ying Huang, Linda Simoni-Wastila

**Affiliations:** 1 Department of Pharmaceutical Health Services Research, University of Maryland Baltimore, Baltimore, MD, 21201, USA

## Background

Although the association between mental health and substance use is well documented, the recent mandate by the Substance Abuse and Mental Health Services Administration to better integrate prevention, treatment, and recovery efforts for both conditions challenges state governments to identify and utilize data to evaluate individuals with co-occurring conditions. At the individual level, it is imperative to understand if either or both disorders exist, as the symptoms of one influence the other. Additionally, treatment approaches vary depending upon the co-existence of substance use and mental health conditions. Population-level understanding of the prevalence of substance use and mental health conditions facilitates the best prioritization, development, and implementation of meaningful prevention and treatment initiatives. This study assesses the utility of Maryland’s substance use treatment admissions data to examine prevalence and trends of co-occurring mental disorders among individuals treated for substance use disorders.

## Materials and methods

Treatment admissions data for fiscal years 2008–2013 from the State of Maryland Automated Record Tracking (SMART) system were used. SMART provides information on admissions to state-supported alcohol and drug treatment programs. Because SMART data are reported at the event level, they may include multiple admissions for the same client. A co-occurring mental disorder is determined by a counselor upon the client’s admission for substance use disorder treatment.

We report findings of: 1) overall trends of co-occurring mental health problems among all admissions; 2) substance-specific trends of prevalence of co-occurring mental health problems; and 3) geographic distribution of the proportion of cases associated with mental disorders by client residence and provider practice in FY2013.

## Results

The total number of treatment admissions in Maryland increased 4.9 percent, from 41482 (FY2008) to 43516 (FY2013). Alcohol, heroin, and marijuana/hashish were the most common substances reported. Admissions with prescription opioid analgesics as the primary substance use disorder more than doubled, from 4.5 percent (FY2008) to 10.3 percent (FY2013). Over the same period, there was a rise (35.0% to 43.8%, respectively) in the percentage of admissions with co-occurring mental health disorders. In FY2013, 64.6 percent of benzodiazepine-related admissions were associated with a co-occurring mental disorder; the corresponding estimates for other substance admissions were 52.4 percent (prescription opioid use); 49.8 percent (heroin use); 44.1 percent of alcohol-related admissions; and 39.0 percent (marijuana use). The prevalence of co-existing mental problems increased across all aforementioned substances between FY2008 and FY2013. The prevalence of co-occurring mental problems varied geographically by both county of client residence and location of the treatment center.

## Conclusions

Substance use treatment admissions indicate how many individuals receive treatment for a substance use disorder and should not be considered the only indicator of the magnitude of substance use. Rather, treatment admissions should be perceived as a consequence stemming from substance use that requires resources. The rising co-occurrence of mental health problems and substance use disorders calls for adaptations in treatment strategies to address the increasing complexity of providing effective treatment. Awareness of variations in the proportion of cases involving a mental health problem at client residence and provider jurisdiction levels can help target prevention, treatment, and mental disorder diagnostic resources more effectively and efficiently.

